# Essential Genes and MiRNA–mRNA Network Contributing to the Pathogenesis of Idiopathic Pulmonary Arterial Hypertension

**DOI:** 10.3389/fcvm.2021.627873

**Published:** 2021-05-05

**Authors:** Shengyu Hao, Pan Jiang, Liang Xie, Guiling Xiang, Zilong Liu, Weiping Hu, Qinhan Wu, Liyan Jiang, Yi Xiao, Shanqun Li

**Affiliations:** Department of Respiratory Medicine, Zhongshan Hospital Affiliated to Fudan University, Shanghai, China

**Keywords:** idiopathic pulmonary arterial hypertension, hub genes, microRNA, lung tissues, GEO

## Abstract

**Background:** Idiopathic pulmonary arterial hypertension (IPAH) is a life-threatening disease. Owing to its high fatality rate and narrow therapeutic options, identification of the pathogenic mechanisms of IPAH is becoming increasingly important.

**Methods:** In our research, we utilized the robust rank aggregation (RRA) method to integrate four eligible pulmonary arterial hypertension (PAH) microarray datasets and identified the significant differentially expressed genes (DEGs) between IPAH and normal samples. Gene Ontology (GO) and Kyoto Encyclopedia of Genes and Genomes (KEGG) pathways were performed to analyze their functions. The interaction network of protein–protein interaction (PPI) was constructed to explore the correlation between these DEGs. The functional modules and hub genes were further identified by the weighted gene coexpression network analysis (WGCNA). Moreover, a miRNA microarray dataset was involved and analyzed to filter differentially expressed miRNAs (DE-miRNAs). Potential target genes of screened DE-miRNAs were predicted and merged with DEGs to explore a miRNA–mRNA network in IPAH. Some hub genes were selected and validated by RT-PCR in lung tissues from the PAH animal model.

**Results:** A total of 260 DEGs, consisting of 183 upregulated and 77 downregulated significant DEGs, were identified, and some of those genes were novel. Their molecular roles in the etiology of IPAH remained vague. The most crucial functional module involved in IPAH is mainly enriched in biological processes, including leukocyte migration, cell chemotaxis, and myeloid leukocyte migration. Construction and analysis of the PPI network showed that CXCL10, CXCL9, CCR1, CX3CR1, CX3CL1, CXCR2, CXCR1, PF4, CCL4L1, and ADORA3 were recognized as top 10 hub genes with high connectivity degrees. WGCNA further identified five main functional modules involved in the pathogenesis of IPAH. Twelve upregulated DE-miRNAs and nine downregulated DE-miRNAs were identified. Among them, four downregulated DEGs and eight upregulated DEGs were supposed to be negatively regulated by three upregulated DE-miRNAs and three downregulated DE-miRNAs, respectively.

**Conclusions:** This study identifies some key and functional coexpression modules involved in IPAH, as well as a potential IPAH-related miRNA–mRNA regulated network. It provides deepening insights into the molecular mechanisms and provides vital clues in seeking novel therapeutic targets for IPAH.

## Background

Pulmonary arterial hypertension (PAH) is a progressive and fatal disease characterized by abnormal cellular apoptotic resistance and vascular remodeling leading to elevated pulmonary pressures and, eventually, right heart failure (1). According to the previous studies, the average time from symptom onset to diagnosis is about 2 years, and the mean survival time of untreated PAH patients is 2.8 years (2). PAH is classified into five major groups, and idiopathic PAH (IPAH) counts for about 40% of cases. It is considered that PAH develops in response to various genetic abnormalities and is triggered by environmental risks (2). Currently, the approved treatments for PAH have been reported to successfully target vasoconstrictive and proliferative mediators, such as prostacyclin receptor agonists, endothelin receptor antagonists, soluble cGMP stimulators, and phosphodiesterase type 5 inhibitors, leading to improved exercise capacity and clinical prognosis (3). However, the individual response to therapy is not uniform, and the patient's condition may be improved, but some have worsened (4–6). As a result, clarifying the gene-specific expression helps explore the pathogenic mechanisms or seek treatment for IPAH.

In the past few decades, microarray analysis has been used for gene-wide expression profiling in lung tissues or peripheral blood from PAH patients or experimental animals to define PAH's pathobiology (6–10). However, the findings are inconsistent between different researches, which may be caused by various analysis platforms, limited sample sizes, different methods, no classification of PAH, and not for IPAH. Although Elinoff et al. performed an integrated analysis of blood expression profiles in PAH (8), the microarray datasets from the lung tissues may be better and more directly reflect the PAH's pathological process. The robust rank aggregation (RRA) method has been used to combine available datasets from independent researches and perform integrative analysis to improve the statistical power and reliability of results (11).

In this study, we aimed to characterize key genes, pathways, and miRNA–mRNA networks related to IPAH. Using RRA method, we integrated four pulmonary microarray datasets of IPAH patients to identify the character gene expression for IPAH. The pathway enrichment analysis of Gene Ontology (GO) and Kyoto Encyclopedia of Genes and Genomes (KEGG), and module alteration in patients with IPAH were conducted to screen hub genes. Furthermore, we utilized a miRNA dataset to identify the possible network of mRNA–miRNA in IPAH. Several hub genes were randomly selected and subsequently verified by RT-PCR in a widely used mice model of PAH.

## Methods

### Microarray Datasets

Two common databases, ArrayExpress (https://www.ebi.ac.uk/arrayexpress/) and Gene Expression Omnibus (GEO) (https://www.ncbi.nlm.nih.gov/geo/), were used to search datasets of IPAH patients. We used the following search terms: pulmonary hypertension or pulmonary arterial hypertension or PAH or PH or idiopathic pulmonary arterial hypertension or IPAH. Two researchers independently screened the two databases and selected relevant datasets based on the same following criteria:

(1) The genome-wide gene or non-coding RNA profiling of IPAH patients and controls was involved in the datasets detected by a high-throughput array or next-generation sequencing.
(2) Samples were from human lung tissues.
(3) Data that could be used for reanalysi, such as raw data or processed data.
(4) Datasets not meeting the above criteria were excluded.

**Figure 1:**
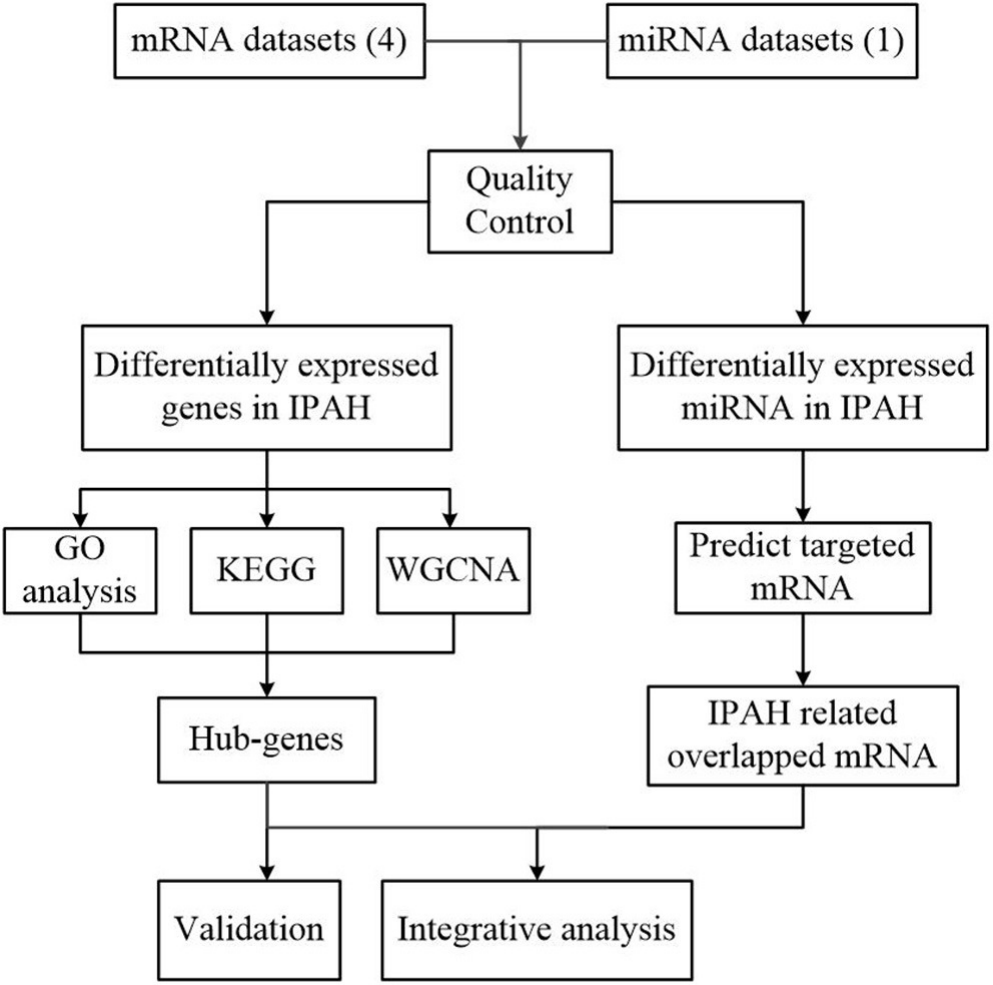
Flowchart of the bioinformatics analysis. MiRNA, microRNA; RRA, robust rank aggregation; IPAH, idiopathic pulmonary arterial hypertension; GO, Gene Ontology; KEGG, Kyoto Encyclopedia of Genes and Genomes; WGCNA, the weighted gene coexpression network analysis.

Our workflow for bioinformatics analysis of publicly available datasets is illustrated in Figure 1.

### Robust Rank Aggregation Analysis and Integration of Datasets

The gene expression profiling was annotated through the corresponding annotation package and the R software (version 3.6.3). The expression data were further normalized by using the “limma” package (Supplementary Figure 1). Then, we performed RRA analysis to rank the upregulated and downregulated expression genes through the R package of “Robust Rank Aggregation” and listed the genes according to their fold changes (11). In the final list, a Bonferroni correction was performed to cut down the false-positive results, and *p*-values that represented the possibility of ranking high were calculated for each gene.

### Functional Enrichment Analysis and the Interaction Network of Protein–Protein Interaction Analysis

We performed GO and KEGG pathway enrichment analyses by using the cluster profile function of R software to match the biological themes of the gene clusters, with a cutoff *p*-value of 0.05. The STRING database (https://string-db.org/cgi/input.pl) was used to establish a PPI network. In the network, the significant Molecular Complex Detection (MCODE) and hub genes were present by using Cytoscape software (version 3.8.0).

### The Weighted Gene Coexpression Network Analysis

To perform weighted gene coexpression network analysis (WGCNA), genes with both *p* < 0.05 and logarithmic fold changes (logFCs) > 0.25 were selected from RRA results. To improve the number of samples and avoid generating less reliable results, we integrated and normalized the four datasets by batch normalization using the “sva” and “limma” package in the R computing environment. We transformed the adjacency matrix into a topological overlap matrix (TOM) and split the genes into different modules based on the TOM-based dissimilarity measure. Key modules were identified by setting the soft-thresholding power of 6 (scale-free R^2^ = 0.9), cut height of 0.25, and minimal module size of 20. We further set gene significance (GS) > 0.3 and module membership (MM) > 0.8 to define the hub genes in WGCNA.

### Identification of Differentially Expressed MiRNAs and Prediction of Target Genes

We downloaded the microRNA expression dataset GSE67597 and identified pulmonary differentially expressed miRNAs (DE-miRNAs) in IPAH patients by using the GEO2R online tool (https://www.ncbi.nlm.nih.gov/geo/geo2r/) with the thresholds of both *p* < 0.05 and |log2FC| > 2. The miRNet database, a useful tool for miRNAs analysis, was used to predict the target genes of DE-miRNAs (12). To visualize the relationship between DE-miRNAs and predicted target mRNAs, we established a miRNA–mRNA network by using Cytoscape (version 3.8.0).

### Animal Models of Pulmonary Arterial Hypertension and Hemodynamic Measurements

Twelve male C57BL/6 mice (8–10 weeks old) were randomly assigned to the following two groups (*n* = 6 per group): CH+SU group: mice were exposed to chronic hypoxia (10% O_2_) for 28 days, weekly receiving a subcutaneous injection of 20 mg/kg of SU5416 (an angiogenesis inhibitor), which was dissolved in a carboxymethylcellulose solution; the normoxic control group (Nor): mice were cultured under normoxic condition and weekly received a subcutaneous injection with an equivalent volume of the dissolvent solution according to their weights, as shown in Supplementary Figure 2. Two groups were provided with food and water *ad libitum*. At the end of the experiment, we used isoflurane to anesthetize the mice and measured their right ventricular systolic pressure (RVSP) by using a Millar pressure transducer catheter through right-sided heart catheterization, as we previously described (13). To evaluate the RV remodeling, we weighed the wall of the RV and the left ventricle plus septum (LV+S) to calculate the ratio of (RV/LV+S).

### Immunohistochemistry, Immunofluorescence, and Imaging

The whole heart and left lung were embedded in paraffin, sliced into 5-μm sections, and then stained with hematoxylin and eosin (H&E) and Masson; and immunofluorescence followed examination with a light microscope (Nikon).

### Real-Time Polymerase Chain Reaction

Total RNA from lungs was isolated using TRIzol, and the extracted RNA was reverse transcribed into complementary DNA (cDNA) using the PrimeScript RT reagent kit (Takara Bio). RT-PCR was carried out using SYBR Green Premix Ex Taq (Takara Bio) to detect the expressions of the genes, which were selected from our analysis. The primer sequences were provided in supplements.

### Statistical Analysis

We used R software and GraphPad Prism 7 to perform statistical analyses, presented data as mean ± standard error, and calculated statistical significance by the Student *t*-test. *p* < 0.05 were considered statistically significant.

## Results

### Pulmonary Arterial Hypertension Microarray Datasets

After filtering ArrayExpress and GEO based on the eligibility criteria, five microarray datasets of PH were finally selected. The information of these datasets was listed, including study country, types of RNA source, GEO accession ID, detection platforms, experiment type, and sample information. The number of IPAH patients in each study ranged from 6 to 32, and the controls ranged from 8 to 25. Finally, a total of 64 IPAH patients and 58 controls were involved in our study (Table 1).

**Table 1:** Summary of those five expression datasets involved in our study.

**Study**	**Country**	**Source types**	**Platform**	**GSE accesion**	**Experiment type**	**Total number**	**Control**	**IPAH**	**PAH**	**Others**
Feghali-Bostwick (2013)	USA	Lung tissues	GPL16221	GSE48149	mRNA	53	9	8	8	SSC-PAH: 10
Mura (2018)	Canada	Lung tissues	GPL6244	GSE113439	mRNA	26	11	6	15	CTD-PAH: 4; CHD-PAH: 4; CTEPH: 1
Stearman (2018)	USA	Lung tissues	GPL6244	GSE117261	mRNA	83	25	32	58	APAH: 17, FPAH: 5; Other: 3; WHO4: 1
Ferhaan (2019)	USA	Lung tissues	GPL6480	GSE15197	mRNA	39	13	18	26	IPF-PH: 8
Gao (2017)	USA	Lung tissues	GPL18402	GSE67597	microRNA	15	8	7	7	None

*PAH, pulmonary arterial hypertension; IPAH, idiopathic PAH; ssc-PAH, SSc-associated PAH; IPF, idiopathic pulmonary fibrosis; IPAH, idiopathic PAH; FPAH, family PAH; CTD-PAH, PAH secondary to connective tissue disease; CHD-PAH, PAH secondary to congenital heart disease; CTEPH, chronic thromboembolic pulmonary hypertension*.

### Identification of the Robust Differentially Expressed Genes by the Robust Rank Aggregation Method

**Figure 2:**
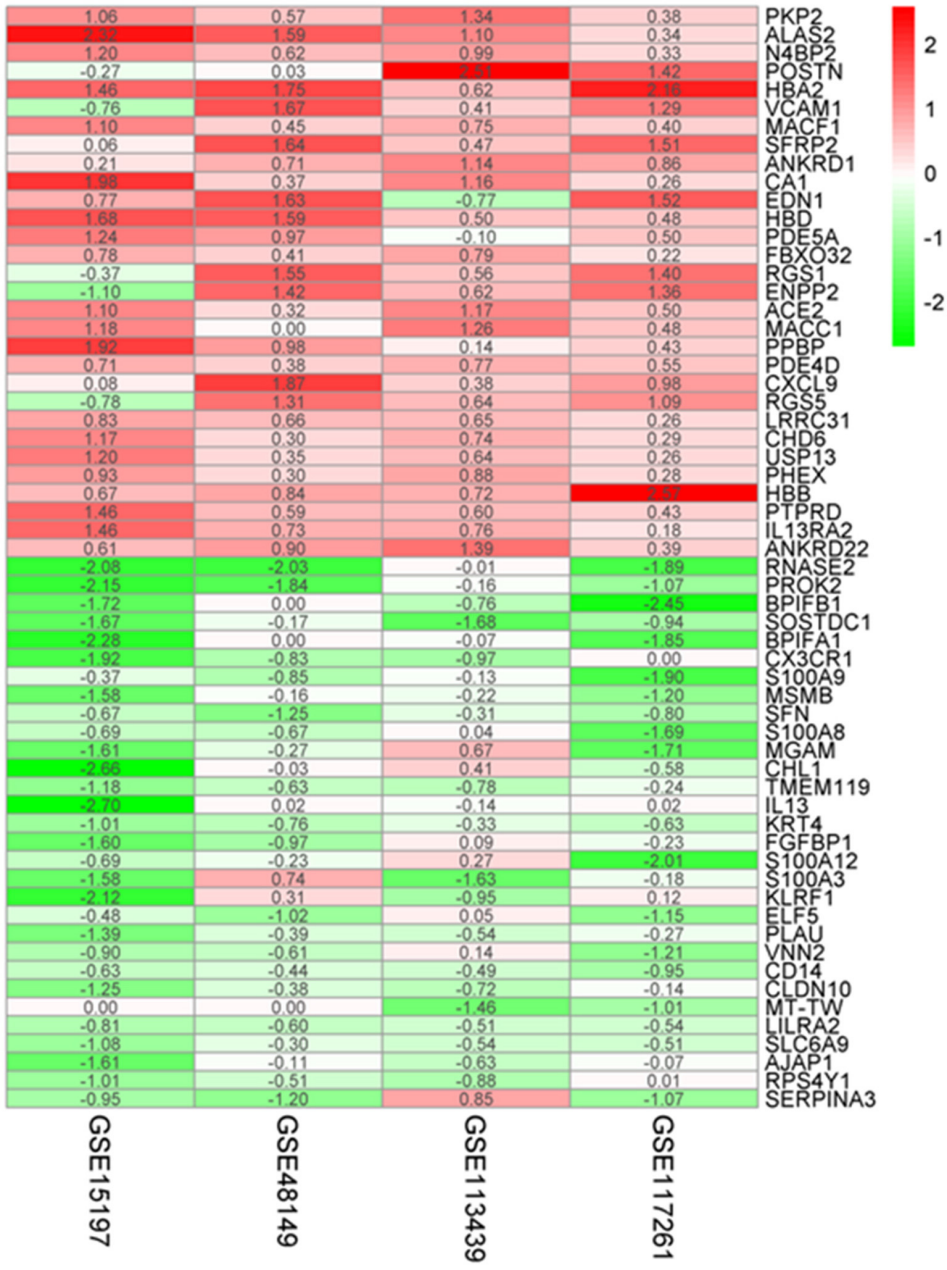
Robust DEGs identified by RRA analysis. Heatmap of the four datasets showing the top 30 upregulated and 30 downregulated DEGs. The horizontal axis indicates the gene name, and the vertical axis represents a dataset. Red indicates that the gene is upregulated in the IPAH patients compared with the controls, and the green represents downregulation. The number in a cell indicates the logFC of each gene in a dataset. DEG, differentially expressed gene; RRA, robust rank aggregation.

A total of 183 upregulated and 77 downregulated differentially expressed genes (DEGs) were successfully identified by integrating four genome-wide gene expression datasets through the RRA method (Supplementary Table 1). Among the upregulated genes, PKP2 was ranked as the first one (*p* = 4.71E−07, adjusted *p* = 1.36E−02), followed by ALAS2 (*p* = 1.11E−06, adjusted *p* = 3.19E−02). Meanwhile, RNASE2 (*p* = 8.94E−10, adjusted *p* = 2.57E−05) and PROK2 (*p* = 2.20E−08, adjusted *p* = 6.33E−04) were ranked as the first and second downregulated genes in RRA analysis. The top 30 upregulated and downregulated genes in IPAH were illustrated by a heatmap (Figure 2). Among these genes, the roles of some ones had been well-explored in PAH, such as interleukin 13 receptor alpha 2 (IL13RA2), angiotensin I converting enzyme 2 (ACE2) (14), and vascular cell adhesion molecule 1 (VCAM1) (15). Notably, some genes were novel, and their functions in PAH had not been researched in published literature, such as hemoglobin alpha 2 (HBA2), frizzled-related protein 2 (SFRP2), and ribonucleases 2 (RNASE2).

### Functional Enrichment Analysis of Differentially Expressed Genes

**Figure 3:**
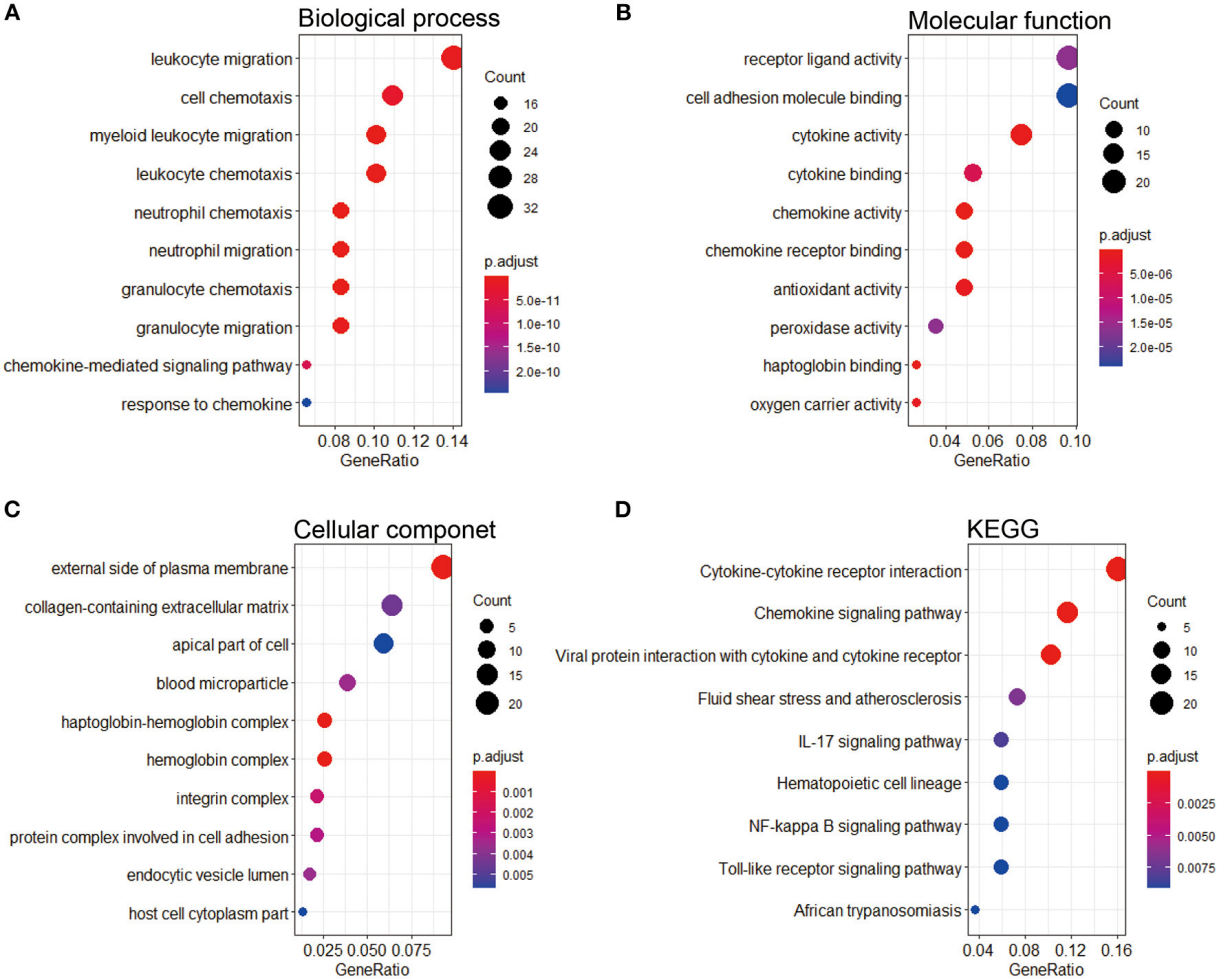
GO and KEGG pathway enrichment of the robust DEGs in IPAH. **(A)** Top 10 biological processes. **(B)** Top 10 molecular functions. **(C)** Top 10 cellular components. **(D)** Top 10 of the KEGG pathway. GO, Gene Ontology; KEGG, Kyoto Encyclopedia of Genes and Genomes; DEG, differentially expressed gene; IPAH, idiopathic pulmonary arterial hypertension.

To research the potential biological pathways involved in IPAH, we performed GO and KEGG to analyze the DEGs. Several enrichment biological processes in GO terms were identified, such as leukocyte migration, cell chemotaxis, and myeloid leukocyte chemotaxis (Figure 3A). In terms of molecular function, receptor-ligand activity was listed as the most significantly GO terms (Figure 3B). Moreover, some cellular component terms were enriched, such as the external side of plasma membrane and collagen-containing extracellular matrix (ECM) (Figure 3C). According to KEGG analysis, we found that the DEGs were mostly associated with cytokine–cytokine receptor interaction, chemokine signaling pathway, viral protein interaction with cytokine and cytokine receptor, fluid shear stress and atherosclerosis, and IL-17 signaling pathway (Figure 3D).

**Figure 4:**
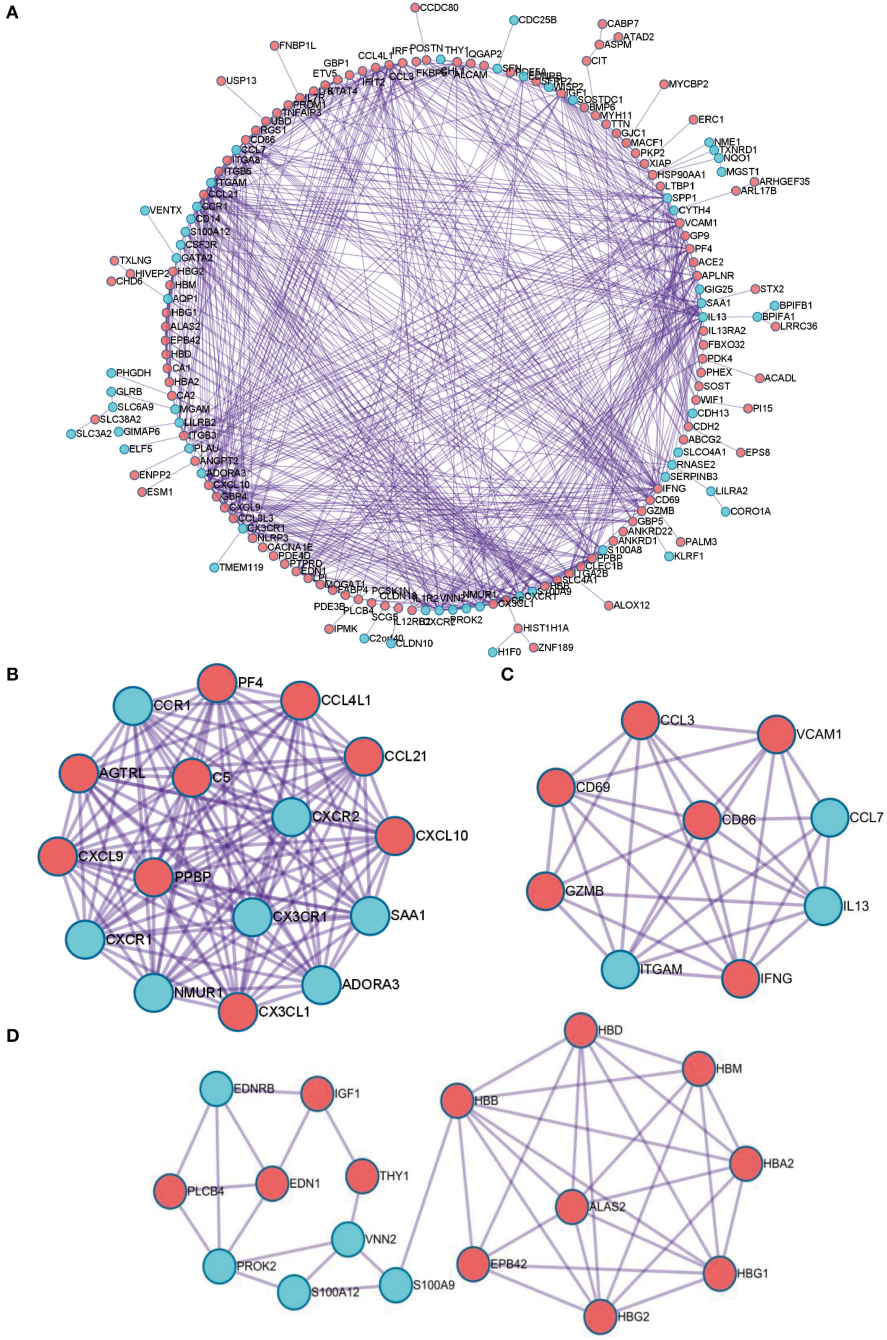
The PPI network of the robust DEGs. **(A)** PPI network of upregulated and downregulated significant genes. **(B–D)** The most significant modules identified through MCODE in Cytoscape software. PPI, protein–protein interaction; DEG, differentially expressed gene.

Next, PPI network of these DEGs was constructed by using the STRING database. The visualization was carried out using Cytoscape software (Figure 4A). MCODE plugin was used to find the top hub genes; the top 3 closely connected modules were identified. The genes in MCODE 1 include CCL21, SAA1, CCL4L1, ADORA3, NMUR1, PPBP, CXCR2, CXCL10, CXCR1, CXCL9, CCR1, AGTRL, PF4, CX3CL1, C5, and CX3CR1, which are associated with Class A/1 (Rhodopsin-like receptors), peptide ligand-binding receptors, and G alpha (i) signaling events; the genes in MCODE 2 are related with positive regulation of leukocyte activation, allograft rejection, and cellular response to interferon-gamma; and the genes in MCODE 3 are associated with oxygen transport and gas transport (Figures 4B–D, Supplementary Table 2).

### Weighted Gene Coexpression Network Analysis

**Figure 5:**
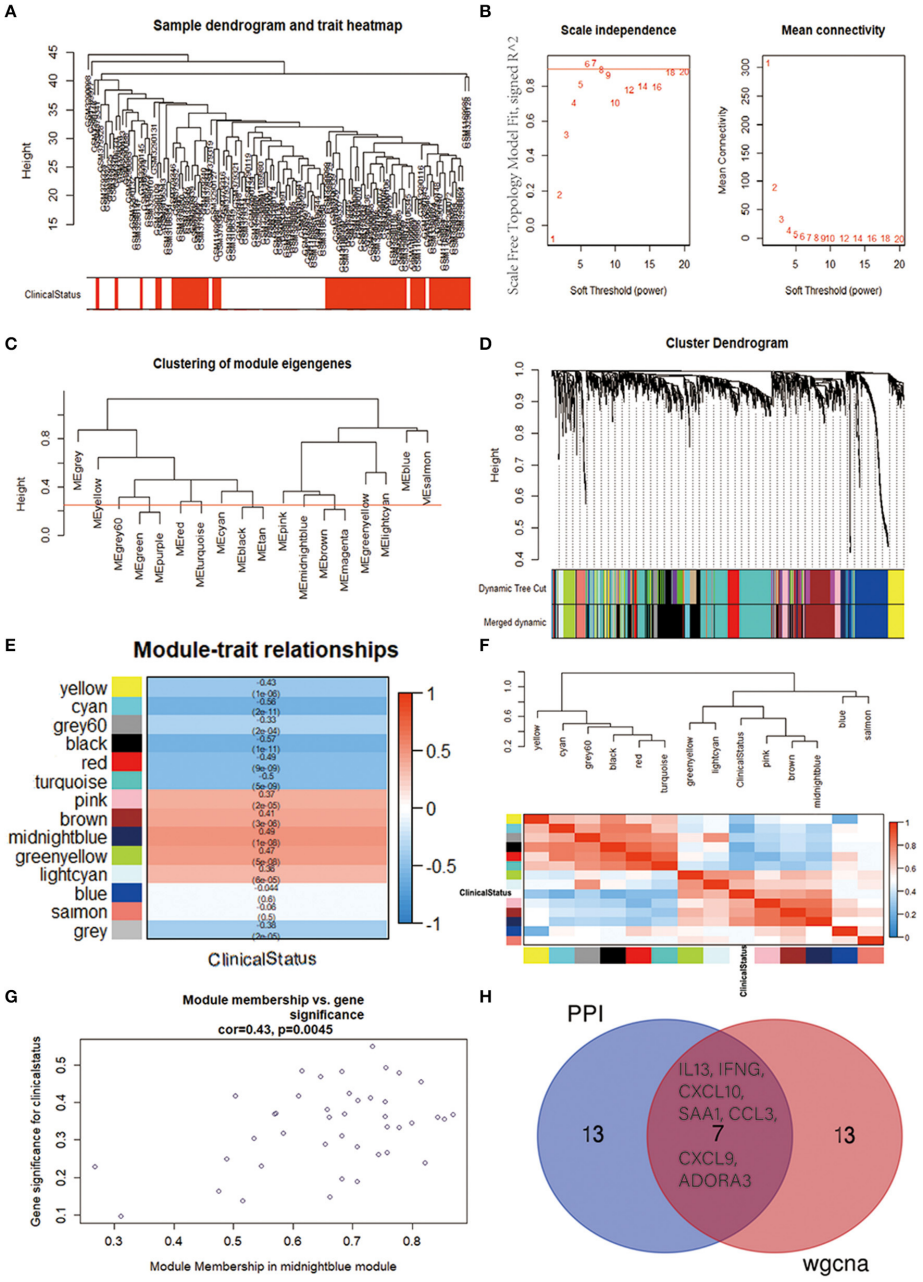
Identification of crucial modules associated with IPAH by WGCNA. **(A)** Clustering dendrograms of genes from bath-normalized four datasets. In the column of clinical status, red indicates IPAH, and white means the control. **(B)** The scale-free fit index (left) and the mean connectivity (right) for various soft-thresholding powers. **(C)** Clustering of module eigengenes. The cut height (red line) was 0.25. **(D)** Dendrogram of the DEGs clustered based on a dissimilarity measure (1—TOM). **(E)** Heatmap showing the relationship between module eigengenes and clinical status. The numbers in each cell means the correlation coefficient and *p*-value. **(F)** Cluster analysis and heatmap of the genes in different modules. Red means a positive correlation, and blue indicates a negative correlation. **(G)** Scatter plot of module eigengenes in the midnight-blue module. **(H)** Venn diagram. The overlap of top 20 genes in the midnight-blue module of WGCNA and top 20 hub genes of PPI analysis. IPAH, idiopathic pulmonary arterial hypertension; WGCNA, weighted gene coexpression network analysis; DEG, differentially expressed gene; TOM, topological overlap matrix; PPI, protein–protein interaction.

To further identify the functional and meaningful modules most associated with IPAH, we utilized WGCNA in these four datasets (Figure 5A). We selected genes with *p* < 0.05 and logFCs > 0.25 from the ranked gene list to cover enough genes in WGCNA. Finally, 14 modules were identified by setting the soft-thresholding power as 6 (scale-free R^2^ = 0.9) and cut height as 0.25 (Figures 5B–D; non-clustering DEGs shown in gray). The correlations between module and IPAH were illustrated in a heatmap, and the midnight-blue module was found to be the most highly correlated module with IPAH (Figures 5E,F). The midnight-blue module contained 42 genes shown in Figure 5G (correlation coefficient = 0.49, *p* = 1E−08G). We selected top 20 hub genes from the midnight-blue module: CXCL10, IFNG, CCL3, CXCL9, VCAM1, ANKRD22, IL13, SAA1, ITGAMT, TNFSF8, NCR1, EOMES, KLRC3, SLC14A1, ADORA3, ZAP70, CD96, CCL5, LCK, and BTLA. Venn diagram displayed the overlap of genes between PPI analysis and WGCNA. The results showed that seven hub genes (INFG, IL13, CXCL10, SAA1, CCL3, CXCL9, and ADORA3) were found in the top 20 hub genes of PPI and top 20 genes of the midnight-blue module (Figure 5H).

### The Potential MiRNA–mRNA Regulatory Network

**Figure 6:**
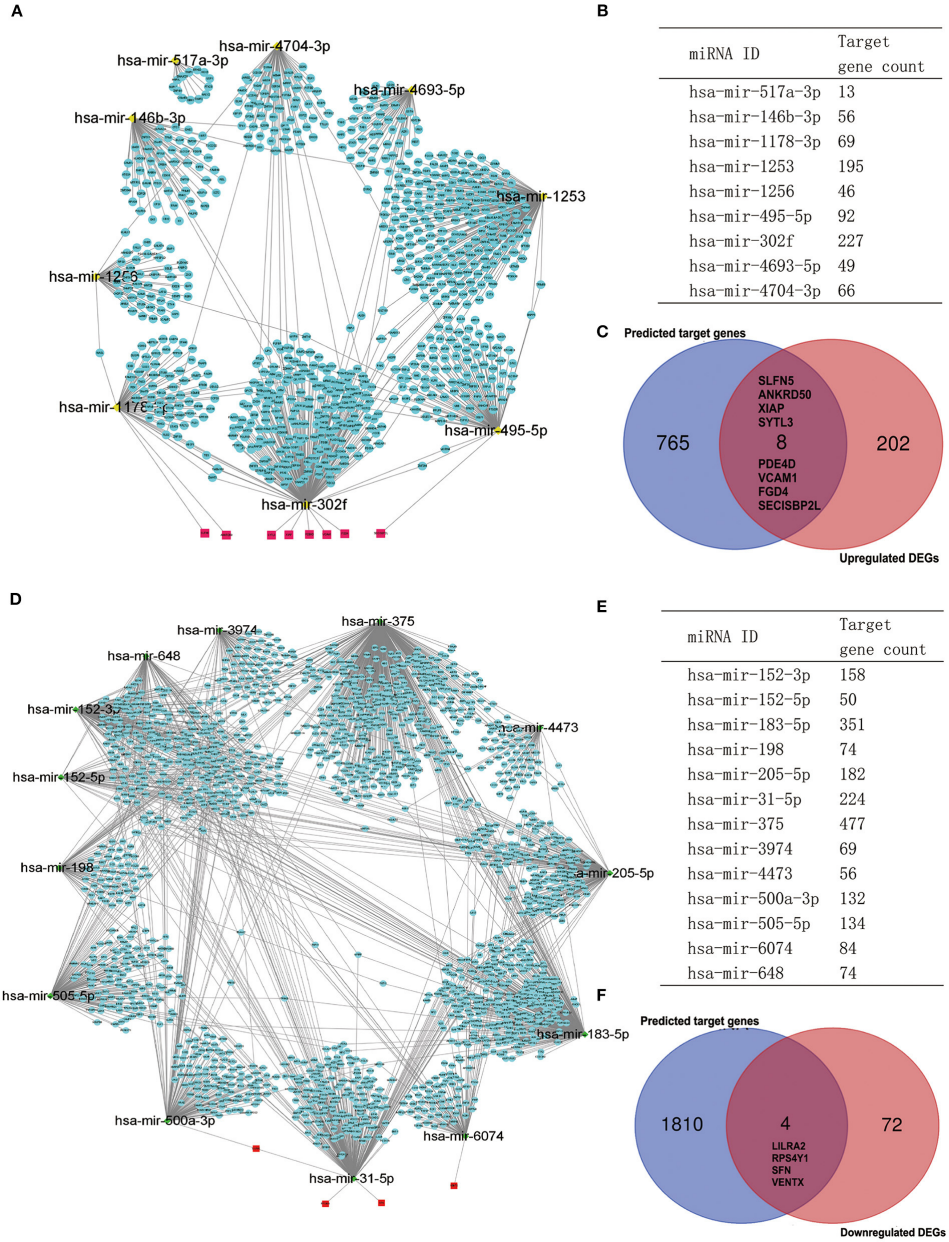
Potential target genes of DE-miRNAs predicted by miRNet database. **(A)** The network of downregulated DE-miRNAs and predicted target genes. **(B)** Predicted target gene count for each downregulated DE-miRNAs. **(C)** The Venn gram of predicted upregulated target genes and upregulated DEGs from RRA analysis. **(D)** The network of upregulated DE-miRNAs and predicted target genes. **(E)** Predicted target gene count for each upregulated DE-miRNAs. **(F)** The Venn gram of predicted downregulated target genes and downregulated DEGs from RRA analysis. DE-miRNAs, differentially expressed miRNAs; DEG, differentially expressed gene; RRA, robust rank aggregation.

**Figure 7:**
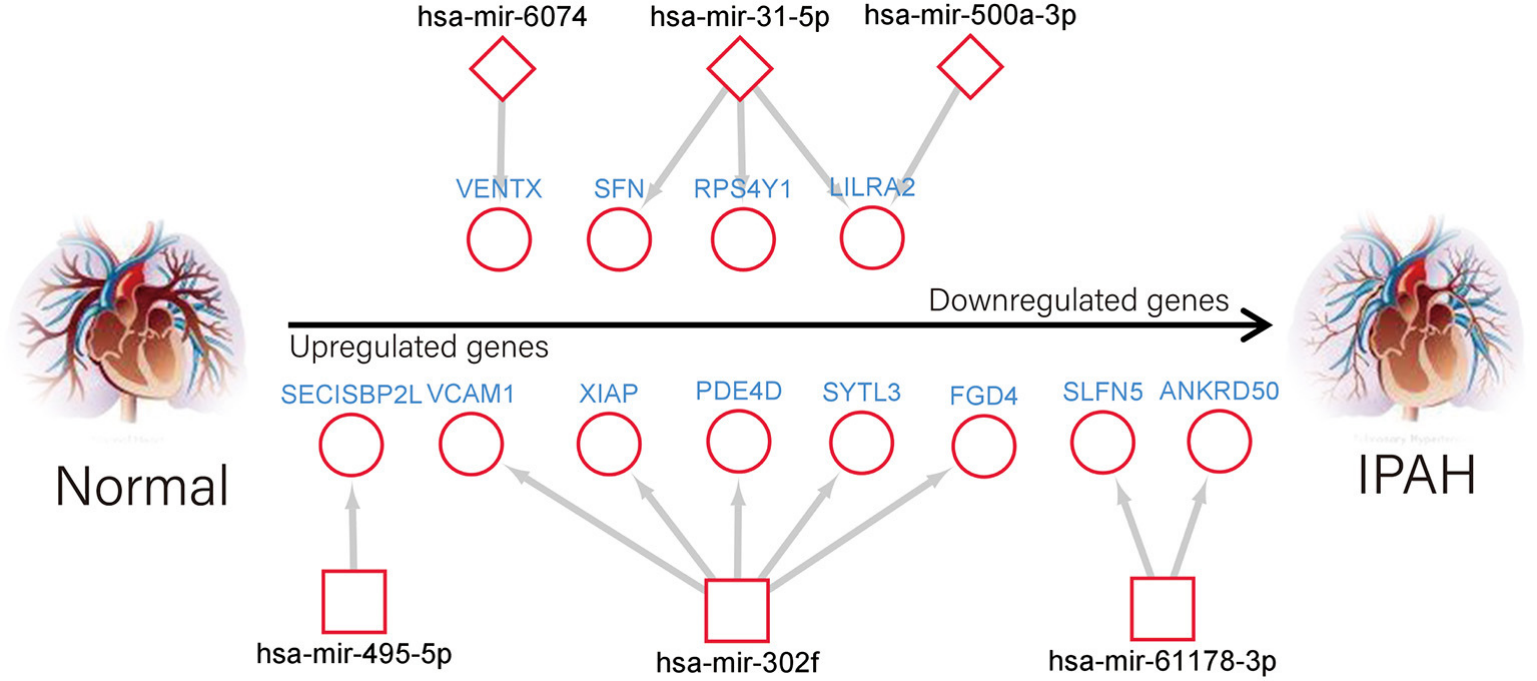
An interactive network of overlapped genes and the upstream miRNAs.

It is widely acknowledged that miRNA exerts an essential effect on PAH by negatively regulating target mRNA. Therefore, we utilized the GEO2R tool to identify the DE-miRNAs in IPAH and predicted the downstream target genes of DE-miRNAs by using the miRNet database. Eventually, we predicted 1,816 and 773 target genes for the 13 upregulated DE-miRNAs and nine downregulated DE-miRNAs, respectively. Furthermore, the upregulated DE-miRNA–target gene network and downregulated DE-miRNA–target gene network were established and presented (Figures 6A,D). The number of target genes for each DE-miRNA is listed in Figures 6B,E. The common genes in DEGs and predicted target genes were identified and shown by the Venn diagram (Figures 6C,F). As a consequence, we found that four target genes (LILRA2, RPS4Y1, SFN, and VENTX) were negatively regulated by increased miRNA-500a-3p, miRNA-31-5p, or miRNA-6074; and eight upregulated genes (SLFN5, ANKRD50, XIAP, SYTL3, PDE4D, VCAM1, FGD4, and SECISBP2L) were the target of miRNA-1178-3p, miRNA-302f, or miRNA-495-5p (Figure 7).

### Validation Through qRT-PCR

**Figure 8:**
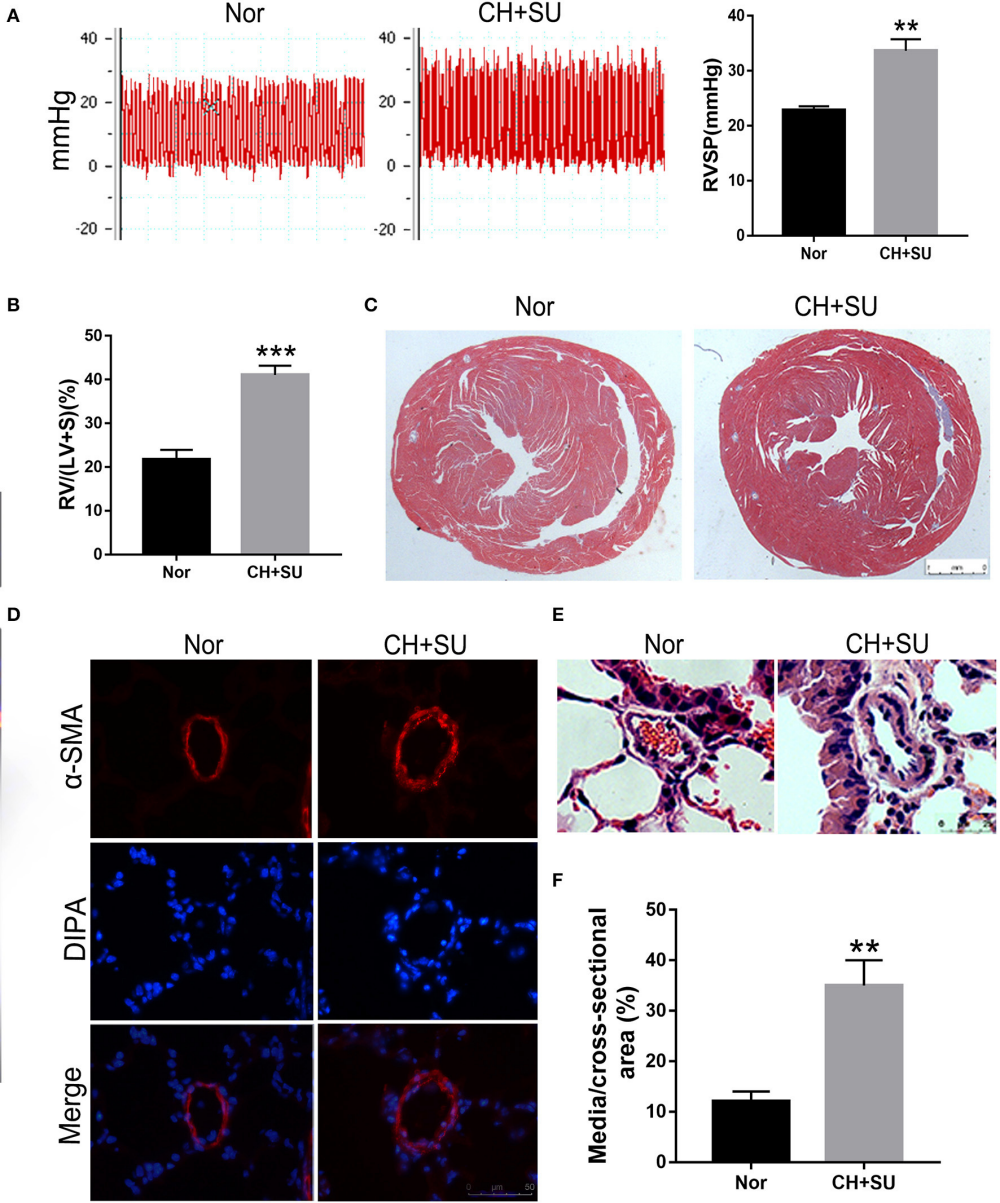
PAH mice model. **(A)** Representative tracing of RVSP in mice after CH+SU or Nor treatment (left) and the mean values of RVSP in the two groups. **(B)** Fulton index (RV/LV+S) in mice after CH+SU or Nor treatment. **(C)** Representative Masson stain images of the hearts from CH+SU or Nor mice. **(D)** Representative α-SMA immunostaining images of lung sections from CH+SU or Nor mice. Bar, 25 μm. **(E)** Representative images of H&E staining of lung sections from CH+SU or Nor mice. Bar, 25 μm. **(F)** The ratio of pulmonary arterial medial thickness to total vessel size for the CH+SU or Nor mice. *n* = 6 in each group. **p < 0.01, ***p < 0.001 vs. Nor group. All graphs are shown as mean ± SEM. PAH, pulmonary arterial hypertension; RVSP, right ventricular systolic pressure.

**Figure 9:**
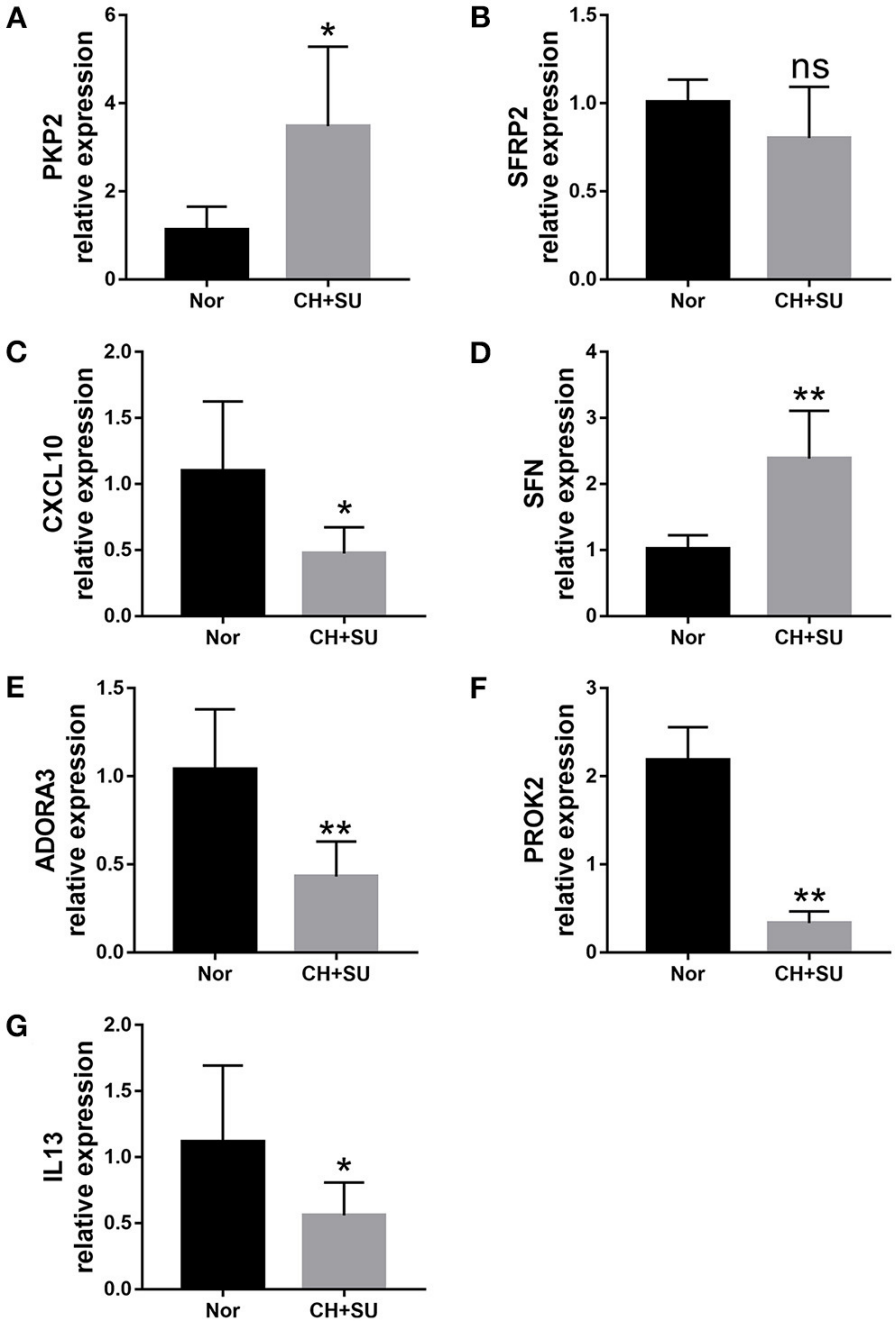
Validation of seven differently expressed key genes through RT-PCR in the lungs from CH+SU or Nor mice. Expression of PKP2 **(A)**, ADORA3 **(B)**, PROK2 **(C)**, IL-13 **(D)**, CXCL10 **(E)**, SFN **(F)**, and SFRP2 **(G)** in CH+SU mice compared with Nor controls. PKP2, plakophilin-2; ADORA3, adenosine A3 receptor; PROK2, prokineticin 2; IL-13, interleukin-13; SFN, stratifin; SFRP2, secreted frizzled-related proteins. *n* = 6 in each group. *p < 0.05, **p < 0.01 vs. Nor group. All graphs are shown as mean ± SEM.

Chronic hypoxia exposure with SU5416 injection is a well-studied and published method to make animal models of PAH. However, there are currently no perfect animal models that replicate entirely human PAH. To investigate whether the pathological changes of IPAH in human also occur in CH+SU mice and to validate the expressions of hub genes found in our analysis, we established PAH model. After a 28-day exposure to CH with the administration of SU5416, mice had a significant elevation in RVSP (Figure 8A) and RV/LV+S compared with those in control mice (Figures 8B,C). Meanwhile, CH+SU treatment resulted in pulmonary vascular remodeling, evidenced by the increased media (Figures 8D–F). Then, we detected the expression of DEGs that interest us in a successfully established PAH mice model. As shown in Figure 9, the expressions of PKP2 (Figure 9A) were significantly upregulated. At the same time, ADORA3, PROK2, and IL-13 (Figures 9B–D) were downregulated in the lungs from CH+SU mice, which were consistent with the results in the datasets of IPAH patients. Meanwhile, we found inconsistent gene expression between the animal model and IPAH patients; for example, CXCL10 (Figure 9E) was downregulated, while SFN (Figure 9F) was upregulated in CH+SU mice, which were contrary to the findings in IPAH patients. The expression of SFRP2 showed no difference between normoxic and CH+SU mice (Figure 9G) but significantly upregulated in IPAH patients based on RRA analysis.

## Discussion

Currently, IPAH is still a progressive, deadly, and incurable disease. Drug therapy has improved symptoms and signs of IPAH, but the mortality remains high, with survival rates estimated at 57–75% at 5 years. The lack of improvement in survival is at least partially attributable to limitations in understanding the mechanisms of IPAH. Although many researchers attempted performing microarray and RNA-seq to screen novel biomarkers and possible therapeutic targets for IPAH, inconsistent results were observed between different studies. To our knowledge, this is the first work to perform RRA, WGCNA, and miRNA dataset to identify significant hub genes and miRNA–mRNA networks related to IPAH. Although Elinoff et al. recently compared gene expression between PAH and control samples for each dataset and obtained DEGs by using R meta-package, the results are based on the blood dataset with no classification of PAH (8). Tao et al. performed a WGCNA between IPAH and control samples; however, this study only contained one blood dataset with 16 IPAH patients and 10 healthy controls (16). To minimize variability, we enrolled lung-tissue datasets from IPAH patients and controls. We involved four qualified PAH datasets into the RRA analysis and observed some significantly upregulated or downregulated DEGs, some of which, such as ACE2 and IL-13, have been reported to play a vital role in PAH pathogenesis. Notably, some DEGs found in our research have never been reported in IPAH, such as PKP2, N4BP2, and ANKRD1. PKP2 was reported to target miR-200b, and deficiency of PKP2 impairs cell–cell contacts, particularly in response to mechanical stress or stretch. N4BP2 was reported to be involved in pulmonary fibrosis (17) and nasopharyngeal carcinoma (18).

It is reported that pathogenic mutations have been observed in ~25% of IPAH without a prior family history of the disease. Among them, the bone morphogenetic protein receptor type II (BMPR2) gene is the single most common causal factor for hereditary cases (19). However, BMPR2 (*p* = 0.17577, logFCs = 0.237) is not listed in the DEGs in our research. The reason may be that not all carriers of BMPR2 mutations developed PAH and other genetic factors acting as modifiers are needed. Besides, other genes involved in the BMP signaling pathway, such as BMP6, BMP5, SMAD9, and SMAD5, are significantly upregulated in our study. The contribution of these genes to PAH is less well-understood but appears to be connected to the capacity to regulate cell growth and survival in the pulmonary arteries.

The pathogenesis of IPAH is complex and heterogeneous. Consistent with published data, the enrichment of these DEGs in GO terms, such as leukocyte migration (20, 21), collagen-containing ECM (22, 23), cytokine activity (24, 25), and antioxidant activity (26), confirms their involvement in the development of IPAH. Besides, enrichment of the identified DEGs in some KEGG pathways, such as the chemokine signaling pathway (24, 25), IL-17 signaling pathway (27, 28), and NF-kappa B signaling pathway (29, 30), also suggests the relevance in IPAH pathogenesis. Based on GO and KEGG analysis results, we suggest that these DEGs are closely associated with immune response and IPAH development. After constructing a coexpression network by PPI and identifying hub genes through WGCNA, we eventually obtained seven hub genes (INFG, IL-13, CXCL10, SAA1, CCL3, CXCL9, and ADORA3). Some of them were demonstrated to exert roles in the pathogenesis of PAH.

In the past few years, increasing researches have suggested that expression changes of miRNAs and downstream target genes are closely associated with the development of PAH. In this present study, we conducted an integrated analysis using miRNA from GEO and DEGs from RRA analysis. Three upregulated DE-miRNAs and three downregulated DE-miRNAs were finally identified. The miRNA–mRNA network screened in our research has been reported in other pulmonary diseases. For example, hsa-mir-31-5p (miR-31-5p) is found to be significantly upregulated in bronchial biopsies from patients with asthma and chronic obstructive pulmonary disease (31), and lung tissues from non-small lung cancer patients (32). These studies have shown that miR-31-5p mediated cell proliferation, apoptosis, migration, and Warburg effect (33), which were also vital for the development of PAH (34). Hsa-miR-500a-3p (miR-500a-3p) is another pivotal miRNA in our integrated network and is upregulated in this study and in the original study (35). For example, miR-500a-3p is strongly associated with the survival of patients with lung adenocarcinoma and is upregulated in various human cancers and non-neoplastic diseases, including liver cancer, gastric cancer, and prostate cancer (36–39).

Experimental models of PAH are critical to gaining a better understanding of pathogenesis and to performing preclinical testing of novel therapeutic approaches. To date, the majority of experimental models are animal models, and the CH+SU mouse model is one of the most commonly used models for PAH. In our research, genes randomly selected from hub genes were validated by RT-PCR in the lungs of CH+SU mice. However, the mRNA expression trends in mice were not wholly consistent with those in IPAH patients. We postulate that this species difference in gene expression may partly explain the inconsistency. Otherwise, the pathological changes of IPAH have not been fully clarified. Animal models can simulate the process of PAH but unable to be entirely consistent with human IPAH. The difference between mice and IPAH patients reminds us that we should be cautious when drawing conclusions based on experimental animal models. There are several limitations in our study, and future research is needed to validate the findings. First, we did not obtain lung tissues from IPAH patients to perform validation in IPAH patients. Second, we did not test the selected genes in other PAH animal models. Finally, assays *in vitro* and *in vivo* will be needed to explore the molecular mechanisms and pathways.

## Conclusions

In this study, we characterized some DEGs, their enrichment pathways, significant gene modules, and miRNA–mRNA network in IPAH by comprehensively utilizing RRA, GO, KEGG, PPI, WGCNA, and other bioinformatics tools. These findings in our work provide new and deepening insights into IPAH. Notably, some of these genes, miRNAs, pathways, and networks are novel, and more work still needs to be done to explore their roles in the pathogenesis of IPAH.

## Data Availability Statement

The original contributions presented in the study are included in the article/Supplementary Material, further inquiries can be directed to the corresponding author/s.

## Ethics Statement

Experiment protocols were approved by the Animal care Committee of Fudan University.

## Author Contributions

SH designed and performed the study. PJ, LX, and GX contributed to the literature research. ZL, QW, WH, YX, LJ, and SL reviewed and edited the manuscript. All authors read and approved the manuscript.

## Conflict of Interest

The authors declare that the research was conducted in the absence of any commercial or financial relationships that could be construed as a potential conflict of interest.
