# Supplementary material for: Essential Genes and MiRNA–mRNA Network Contributing to the Pathogenesis of Idiopathic Pulmonary Arterial Hypertension
Source: Front Cardiovasc Med. 2021 May 5;8:627873. doi: 10.3389/fcvm.2021.627873 (PMC8133434; doi:10.3389/fcvm.2021.627873)
Supplement: Supplementary file 1 [file Data_Sheet_1.docx]

Supplementary Figure 1


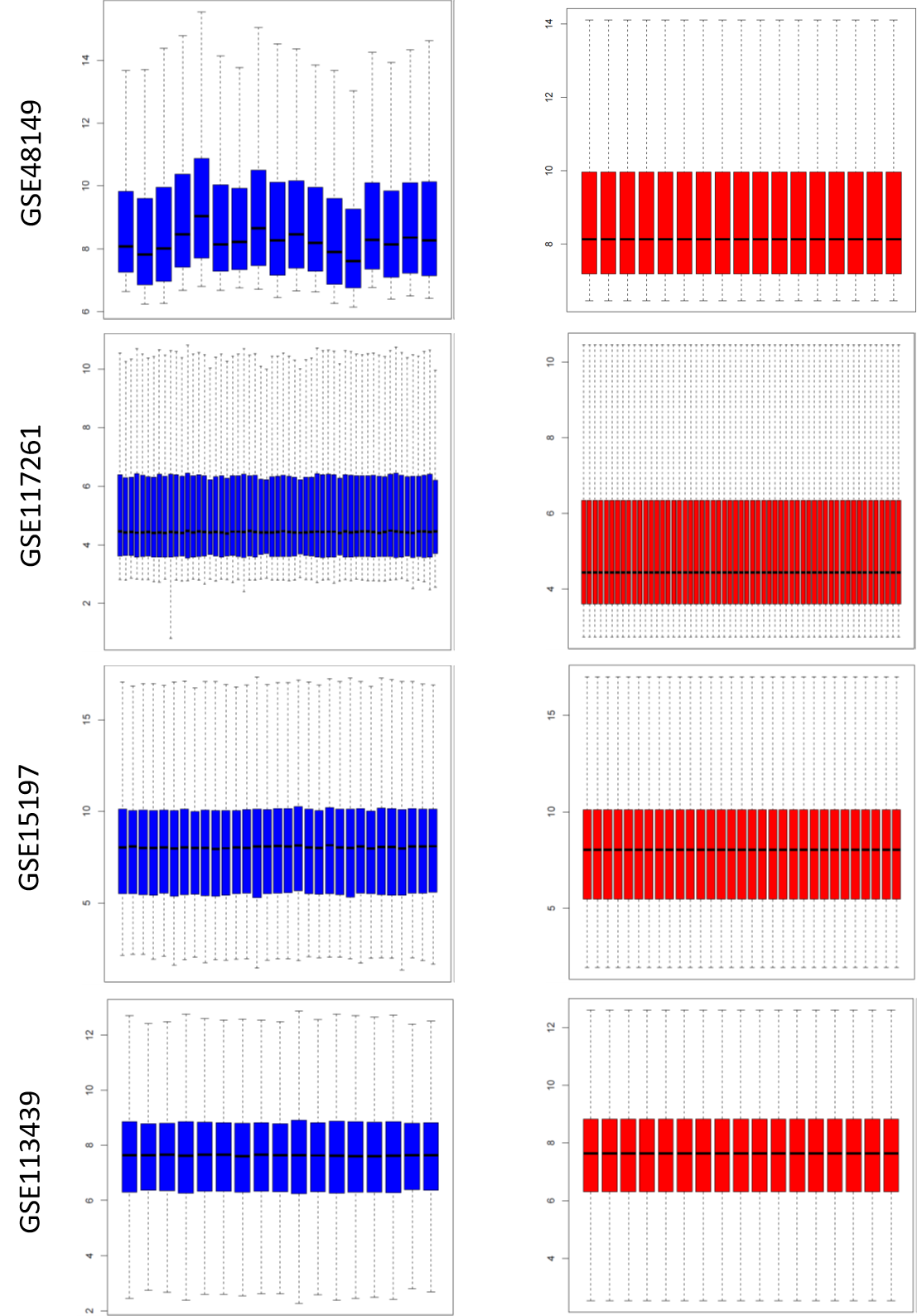


Normalization of raw data in Gene Expression Omnibus database. Blue represents raw data and red represents data after normalization.

Supplementary Figure 2.


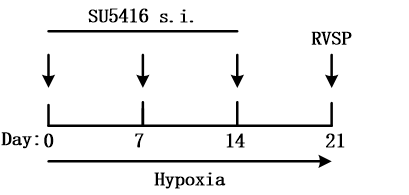


Protocol for CH+SU induced PAH in mice.

The primer of mRNA provided as follows:

| ADORA3 | Forward primer | AAGGTGAAATCAGGTGTTGAGC |
| --- | --- | --- |
|  | Reverse primer | AGGCAATAATGTTGCACGAGT |
| Pkp2 | Forward primer | GCCGAGTGTGGCTACATCC |
|  | Reverse primer | CTGCTGGTTCGGTGAAGGTT |
| CXCL10 | Forward primer | CCAAGTGCTGCCGTCATTTTC |
|  | Reverse primer | GGCTCGCAGGGATGATTTCAA |
| SFN | Forward primer | GTGTGTGCGACACCGTACT |
|  | Reverse primer | CTCGGCTAGGTAGCGGTAG |
| SFRP2 | Forward primer | CGTGGGCTCTTCCTCTTCG |
|  | Reverse primer | ATGTTCTGGTACTCGATGCCG |
| PROK2 | Forward primer | GCCCCGCTACTGCTACTTC |
|  | Reverse primer | CCGCACTGAGAGTCCTTGTC |
| IL-13 | Forward primer | CCTGGCTCTTGCTTGCCTT |
|  | Reverse primer | GGTCTTGTGTGATGTTGCTCA |

Supplementary Table 1. DEGs by RRA

| Name | logFC |  | Name | logFC |  | Name | logFC |  |
| --- | --- | --- | --- | --- | --- | --- | --- | --- |
| PKP2 | 0.836948 | up | LOC649853 | 0.740365 | up | C16orf11 | 0.561337 | up |
| ALAS2 | 1.338937 | up | LRRC17 | 0.511006 | up | RHBDL1 | 0.543504 | up |
| N4BP2 | 0.785303 | up | THY1 | 0.571377 | up | IPMK | 0.524342 | up |
| POSTN | 0.924967 | up | FMO5 | 0.590881 | up | PYY2 | 0.508639 | up |
| HBA2 | 1.497057 | up | SYTL3 | 0.569474 | up | ERC1 | 0.535841 | up |
| VCAM1 | 0.65449 | up | ABCG2 | 0.595972 | up | DKFZP434A062 | 0.525208 | up |
| MACF1 | 0.675482 | up | HBM | 0.672676 | up | STX2 | 0.520584 | up |
| SFRP2 | 0.918464 | up | ATAD2 | 0.507135 | up | XIAP | 0.573464 | up |
| CA1 | 0.943118 | up | IFNG | 0.870634 | up | CD86 | 0.57781 | up |
| ANKRD1 | 0.729015 | up | IFIT2 | 0.523261 | up | FOXN2 | 0.596528 | up |
| EDN1 | 0.790396 | up | ITGA2B | 0.581982 | up | CIT | 0.511641 | up |
| HBD | 1.061025 | up | NCKAP5 | 0.59818 | up | RNASE2 | -1.50167 | down |
| PDE5A | 0.652174 | up | CD5L | 0.501532 | up | PROK2 | -1.30308 | down |
| FBXO32 | 0.550321 | up | WIF1 | 0.778015 | up | SOSTDC1 | -1.11483 | down |
| RGS1 | 0.785382 | up | RAB39B | 0.503622 | up | CX3CR1 | -0.92975 | down |
| ENPP2 | 0.575109 | up | CABP7 | 0.748127 | up | BPIFA1 | -1.04857 | down |
| ACE2 | 0.772499 | up | TTN | 0.650179 | up | TXNRD1 | -0.50777 | down |
| MACC1 | 0.730483 | up | ALOX12 | 0.547469 | up | TMEM119 | -0.70663 | down |
| PPBP | 0.86873 | up | C5 | 0.519823 | up | PLAU | -0.6495 | down |
| PDE4D | 0.603703 | up | RPA4 | 0.554707 | up | LILRA2 | -0.61291 | down |
| CXCL9 | 0.82855 | up | POU3F3 | 0.721604 | up | SERPINA3 | -0.59623 | down |
| RGS5 | 0.564399 | up | CCL3L3 | 0.645887 | up | BPIFB1 | -1.23155 | down |
| LRRC31 | 0.600155 | up | HIVEP2 | 0.522129 | up | ELF5 | -0.65057 | down |
| CHD6 | 0.626552 | up | GSTT1 | 0.747096 | up | ADORA3 | -0.5758 | down |
| USP13 | 0.614437 | up | GPR78 | 0.711123 | up | SLC6A9 | -0.60969 | down |
| PHEX | 0.597135 | up | AGTRL1 | 0.579716 | up | MT-TW | -0.6182 | down |
| HBB | 1.201805 | up | CCDC141 | 0.500048 | up | MS4A15 | -0.50137 | down |
| PTPRD | 0.769404 | up | IL12RB2 | 0.519138 | up | GIMAP6 | -0.54701 | down |
| IL13RA2 | 0.783712 | up | CLDN18 | 0.616404 | up | CORO1A | -0.53933 | down |
| ANKRD22 | 0.821739 | up | JMJD1C | 0.504999 | up | PHGDH | -0.51552 | down |
| PDE8B | 0.760499 | up | NEUROG3 | 0.651993 | up | SLC3A2 | -0.57654 | down |
| ECM2 | 0.554587 | up | CCL4L1 | 0.566269 | up | S100A9 | -0.81278 | down |
| CD69 | 0.51672 | up | CX3CL1 | 0.546082 | up | GATA2 | -0.51554 | down |
| ZNF189 | 0.51218 | up | ARL17B | 0.514698 | up | CD14 | -0.62898 | down |
| GBP5 | 0.870255 | up | MAFA | 0.535928 | up | MGAM | -0.73121 | down |
| FNBP1L | 0.746469 | up | SOST | 0.717765 | up | NQO1 | -0.58846 | down |
| ETV5 | 0.619072 | up | CCL3 | 0.501729 | up | SFN | -0.75656 | down |
| GBP4 | 0.625184 | up | LTBP1 | 0.530855 | up | FGFBP1 | -0.67671 | down |
| HBG2 | 0.728785 | up | CMTM5 | 0.602771 | up | IL1R2 | -0.51027 | down |
| IQGAP2 | 0.800301 | up | ESM1 | 0.555588 | up | RPS4Y1 | -0.59976 | down |
| TNFAIP3 | 0.886959 | up | IGF1 | 0.529349 | up | KRT4 | -0.68411 | down |
| CGNL1 | 0.590769 | up | HSP90AB3P | 0.528328 | up | S100A3 | -0.66161 | down |
| BMP6 | 0.613331 | up | EGFL6 | 0.550711 | up | MSMB | -0.78878 | down |
| CXCL10 | 0.846103 | up | FKBP5 | 0.556443 | up | CLDN10 | -0.62369 | down |
| CDH2 | 0.58857 | up | PF4 | 0.714711 | up | WISP2 | -0.5867 | down |
| CNOT6L | 0.536366 | up | IFITM5 | 0.620483 | up | KLRF1 | -0.6607 | down |
| HBG1 | 0.847299 | up | GJC1 | 0.603117 | up | LILRA3 | -0.53685 | down |
| UBD | 0.784184 | up | ARHGEF38 | 0.542464 | up | H1F0 | -0.55149 | down |
| ANGPT2 | 0.60877 | up | EPB42 | 0.522797 | up | IL13 | -0.69995 | down |
| PLCB4 | 0.532431 | up | XIST | 0.643407 | up | SCARNA4 | -0.52991 | down |
| EHF | 0.606538 | up | IL7R | 0.550861 | up | VNN2 | -0.6425 | down |
| TXLNG | 0.614314 | up | CLEC1B | 0.676161 | up | EDNRB | -0.52327 | down |
| MYH11 | 0.560126 | up | NEUROG1 | 0.548014 | up | AQP1 | -0.54164 | down |
| ANKRD50 | 0.598971 | up | MYCBP2 | 0.505507 | up | CHL1 | -0.7133 | down |
| ITGB3 | 0.502899 | up | HIPK2 | 0.533604 | up | LILRB2 | -0.53078 | down |
| ZNF148 | 0.607357 | up | NBPF9 | 0.544238 | up | S100A8 | -0.74963 | down |
| ITK | 0.528799 | up | FLJ22184 | 0.589796 | up | SPP1 | -0.53005 | down |
| CA2 | 0.700165 | up | COX6A2 | 0.588718 | up | MGST1 | -0.55396 | down |
| PI15 | 0.668102 | up | CD300LG | 0.564044 | up | CDC25B | -0.51944 | down |
| ZNF234 | 0.551485 | up | ZNF654 | 0.517485 | up | S100A12 | -0.66496 | down |
| TFCP2L1 | 0.6669 | up | GPR153 | 0.515076 | up | TSPAN7 | -0.54283 | down |
| STAT4 | 0.632026 | up | IRF1 | 0.501666 | up | TCTEX1D1 | -0.54058 | down |
| ZNF682 | 0.504593 | up | PCSK1N | 0.565935 | up | CALCRL | -0.51907 | down |
| PDE3B | 0.503982 | up | ALCAM | 0.506958 | up | CCR1 | -0.5098 | down |
| ACADL | 0.65118 | up | C9orf62 | 0.609797 | up | SAA1 | -0.51673 | down |
| PRDM1 | 0.532379 | up | HSP90AA1 | 0.601981 | up | NME1 | -0.50287 | down |
| SECISBP2L | 0.642228 | up | LOC145694 | 0.59628 | up | SLCO4A1 | -0.53671 | down |
| RNF150 | 0.517058 | up | MOGAT1 | 0.723594 | up | CYTH4 | -0.53547 | down |
| CACNA1E | 0.855778 | up | LOC100290566 | 0.595141 | up | MIR100HG | -0.55508 | down |
| FMO2 | 0.639368 | up | HIST1H1A | 0.557392 | up | AJAP1 | -0.60385 | down |
| GZMB | 0.838943 | up | ASPM | 0.544154 | up | CSF3R | -0.5654 | down |
| LRRC36 | 0.577279 | up | NLRP3 | 0.506352 | up | SERPINB3 | -0.54947 | down |
| ITGA8 | 0.675962 | up | KRT1 | 0.621001 | up | CDH13 | -0.54181 | down |
| PDK4 | 0.784269 | up | SCG5 | 0.516475 | up | VENTX | -0.53939 | down |
| XRN1 | 0.577341 | up | CCDC80 | 0.530186 | up | ITGAM | -0.54788 | down |
| SLC4A1 | 0.530644 | up | EN2 | 0.575896 | up | TMEM130 | -0.56143 | down |
| GBP1 | 0.589851 | up | LOC393078 | 0.58003 | up | NMUR1 | -0.51333 | down |
| SLC38A2 | 0.569463 | up | EPS8 | 0.5132 | up | POM121L9P | -0.51784 | down |
| FABP4 | 0.611867 | up | CMIP | 0.581398 | up | CYP4Z1 | -0.57839 | down |
| ITGB6 | 0.543509 | up | GP9 | 0.582607 | up | CCL7 | -0.52084 | down |
| COL14A1 | 0.757926 | up | GPR150 | 0.527447 | up | SHISA3 | -0.5147 | down |
| FGD4 | 0.706499 | up | ZSCAN10 | 0.559293 | up | CXCR1 | -0.51938 | down |
| CCL21 | 0.607399 | up | SYNGR4 | 0.536221 | up | C2orf40 | -0.52809 | down |
| LPL | 0.572859 | up | SLFN5 | 0.521108 | up | USP9Y | -0.54413 | down |
| ARHGEF35 | 0.940873 | up | PALM3 | 0.57825 | up | BTNL3 | -0.52739 | down |
| C10orf10 | 0.812553 | up | MOP-1 | 0.63882 | up | CXCR2 | -0.50355 | down |

Supplementary Table 2. MCODE

|  | **Genes** | **Description** | **Log10(P)** |
| --- | --- | --- | --- |
| MCODE_1 | CCL21, SAA1, CCL4L1, ADORA3, NMUR1, PPBP, CXCR2, CXCL10, CXCR1, CXCL9, CCR1, AGTRL, PF4, CX3CL1, C5, CX3CR1 | Class A/1 (Rhodopsin-like receptors) | -22.4 |
|  |  | Peptide ligand-binding receptors | -21.9 |
|  |  | G alpha (i) signalling events | -21.4 |
| MCODE_2 | CCL3, CD86, CCL7, GZMB, CD69, VCAM1, IL13, ITGAM, IFNG | positive regulation of leukocyte activation | -8.86 |
|  |  | Allograft rejection | -6.52 |
|  |  | cellular response to interferon-gamma | -6.42 |
| MCODE_3 | PROK2, S100A9，ALAS2, S100A12,PLCB4, THY1, IGF1, HBM, HBD, HBA2, HBB, EDNRB, HBG1, EDN1, VNN2, EPB42, HBG2 | oxygen transport | -13 |
|  |  | gas transport | -12.6 |
|  |  | hydrogen peroxide catabolic process | -11.5 |
| Top 20 in network string interaction ranked by MCC method | CXCL10, CXCL9, CCR1, CX3CR1, CX3CL1, CXCR2, CXCR1, PF4, CCL4L1, ADORA3, CCL21, SAA1, NMUR1, APLNR, C5, CCL3, ITGAM, IL13, IFNG | | |

The parameters of MCODE are as follows: the value of degree cutoff is 2; the cluster finding is haircut, the value of node score cutoff is 0.2, the K-Core: is 2, and the max. depth is 100.
